# Stress-Axis Regulation by Vitamin D_3_ in Multiple Sclerosis

**DOI:** 10.3389/fneur.2018.00263

**Published:** 2018-04-26

**Authors:** Linda Rolf, Jan Damoiseaux, Inge Huitinga, Dorien Kimenai, Jody van den Ouweland, Raymond Hupperts, Joost Smolders

**Affiliations:** ^1^School for Mental Health and Neuroscience, Maastricht University, Maastricht, Netherlands; ^2^Zuyderland Medical Center, Academic MS Center Limburg, Sittard, Netherlands; ^3^Maastricht University Medical Center, Central Diagnostic Laboratory, Maastricht, Netherlands; ^4^Netherlands Institute for Neuroscience, Neuroimmunology Research Group, Amsterdam, Netherlands; ^5^Canisius Wilhelmina Hospital, Department of Clinical Chemistry, Nijmegen, Netherlands; ^6^Canisius Wilhelmina Hospital, Department of Neurology, Nijmegen, Netherlands

**Keywords:** Cortisol, HPA-axis, multiple sclerosis, supplementation, vitamin D

## Abstract

**Introduction:**

Multiple sclerosis (MS) has been associated with both a poor vitamin D status and hyperactivity of the hypothalamus–pituitary–adrenal (HPA) axis. Since nuclear receptor ligands may regulate each other, we explored the association of vitamin D_3_ supplements with circadian cortisol levels in a double-blind and placebo-controlled supplementation study.

**Methods:**

Female patients with relapsing-remitting MS received vitamin D_3_ supplements (4,000 IU/day; *n* = 22) or placebo (*n* = 19) during 16 weeks. Salivary cortisol levels, repeatedly measured during the day, and serum 25(OH)D levels were assessed before (T0) and after (T1) this treatment period.

**Results:**

Median 25(OH)D levels at T1 were 139.9 (interquartile range 123.5–161.2) and 74.5 nmol/L (58.6–88.1) in the vitamin D_3_ and placebo group, respectively (*p* < 0.001). Comparisons within and between groups showed no differences in area under the curve (AUC) and slope of the cortisol day curve. Although the AUC of the cortisol awakening response (CAR, sampling each 15 min the first hour after awakening) showed a reduction over time in the vitamin D_3_ group [39.16 nmol/L (27.41–42.07) at T0 to 33.37 nmol/L (26.75–38.08) at T1] compared to the placebo group [33.90 nmol/L (25.92–44.61) at T0 to 35.00 nmol/L (25.46–49.23) at T1; *p* = 0.044], there was no significant difference in AUC of CAR at T1 corrected for baseline AUC of CAR (*p* = 0.066).

**Conclusion:**

Suppression of HPA-axis activity by vitamin D_3_ supplements in non-depressed MS patients may be best reflected by CAR as primary outcome measure. Further studies should address this interaction and its potential implications for the disease course of MS.

**Registration:**

This study was registered on ClinicalTrials.gov (NCT02096133) and EudraCT (2014-000728-97).

## Introduction

A poor vitamin D_3_ status is frequently encountered in patients with multiple sclerosis (MS) (1), and has been associated with adverse disease outcomes regarding clinical exacerbations of disease (2), disease activity as reflected by MRI (3, 4), and disability progression in the earliest but not later stages of MS (5, 6). Additionally, a low vitamin D_3_ status was found in patients with high depression scores (7). Clinical trials investigate the effect of vitamin D_3_ supplements on disease outcomes, and suggest a positive effect on MRI markers of disease (8).

Vitamin D_3_ is a nuclear receptor ligand, which shows several similarities with other nuclear receptor ligands which have been studied in MS (9). These include corticosteroids, which are the main effector molecules of the hypothalamus–pituitary–adrenal (HPA) or stress-axis. For decades exacerbations of relapsing-remitting MS (RRMS) are treated with pulsed high-dose methylprednisolone therapy (10). Hyperactivation of the stress-axis, as demonstrated by elevated endogenous levels of cortisol and suppression and/or stimulation tests (11–15), has been found in MS. Most importantly, higher cortisol levels or disturbed suppression-tests have been associated with a more severe disease course of MS (16, 17), This makes cortisol a potential relevant biomarker to be affected by therapies.

Several observations suggest that vitamin D may regulate the stress-axis. In hippocampal cell cultures, glucocorticoid-induced transcription and cytotoxicity were suppressed by vitamin D_3_ (18). In the human CNS, the most intense staining for vitamin D receptor and activating enzyme was found in the hypothalamus, including the paraventricular nucleus (18, 19). We showed that corticotrophin releasing hormone-positive neurons in this nucleus, being part of the stress-axis, also stained positive for vitamin D 24-hydroxylase (19), and therefore are likely vitamin D responsive. Whether the stress-axis in MS is regulated by vitamin D *in vivo* in MS is unknown. Therefore, we investigated in an explorative study whether supplementation of vitamin D_3_ is associated with a suppression of saliva cortisol levels in patients with RRMS.

## Materials and Methods

### Study Design and Setting

This was a multi-center, double-blind, placebo-controlled supplementation study. Recruitment and study visits took place in three hospitals in the Netherlands [Zuyderland medical center, locations Sittard and Heerlen, and Canisius Wilhelmina Hospital (CWZ)] between October 2014 and November 2016 (20).

The study was initially designed as a randomized controlled clinical trial to measure the effects of vitamin D_3_ supplements on primary outcome area under the curve (AUC) of cortisol day curves (CDC) in RRMS patients in the context of depressive complaints. The initially projected sample size was calculated based on findings from Gold et al. (21), showing a difference in AUC of CDC between RRMS patients with and without comorbid depression. Due to inclusion difficulties, the aimed number of 80 participants (2× *n* = 32 with 20–25% loss to follow-up) was not reached and the study was prematurely terminated. We used the sampled material to explore an association of vitamin D_3_ supplements with characteristics of the stress-axis.

### Participants

All participants were female patients with RRMS, according to the McDonald criteria (22). Other inclusion criteria were age ≥18 years, and treatment with injectable or oral disease modifying drugs (DMD; interferon-beta, glatiramer acetate, dimethyl fumarate, teriflunomide, or fingolimod) or no DMD treatment, without changes in therapy in the last 3 months. Also, participants had to be pre-menopausal with a perceptible menstrual cycle. Oral contraception was allowed. Exclusion criteria were all contraindications for vitamin D supplementation according to summary of products of the study medication, relapse within 6 weeks prior to study initiation, use of systemic glucocorticoids within 8 weeks prior to study initiation, use of vitamin D supplements >1,000 IU/day, current (treatment for) major depression, pregnancy, and glucocorticoid treatment for relapses during the trial.

### Intervention

Participants were randomly allocated in a 1:1 ratio to either of the intervention groups, being the vitamin D_3_ group or the placebo group. Participants allocated to the vitamin D_3_ group received cholecalciferol drops (Vigantol Oil, Merck, Darmstadt, Germany) dosed at 4,000 IU/day and patients in the placebo group received drops containing only medium-chain triglycerides (Merck) as an additive to ongoing other therapies. Physical appearances of the solutions and packages were identical, and labeling performed by the Zuyderland pharmacy was such that the blinded nature of the trial was protected.

### Randomization

Stratified random sampling was performed by the Zuyderland pharmacy using a predefined blocked randomization list (Sealed Envelope Ltd.; blocks of four), and participants were stratified by EDSS (≤3.5 or >3.5) and depression scores (<3, 3–6, 6–9, or >9). Only the staff of the pharmacy, not directly involved in the trial conduct, had the stratification-randomization scheme. Study personnel, participants, and care providers were blinded to the study interventions.

### Outcome Variables

The main HPA-axis related outcomes of this study were the change in the AUC of the CDC between the two intervention groups, changes in slope of the CDC, and the AUC of the cortisol awakening response (CAR). Timepoints of sampling are specified below. Furthermore, serum and urine calcium levels were monitored as safety parameters. Since the study was initialized in the context of depressive complaints, and depression and fatigue can be relevant confounders of HPA-axis activity, depression scores were obtained by the Hospital Anxiety and Depression Scale (HADS), of which the depression subscale (HADS-D) was used, and fatigue was analyzed with the Fatigue severity scale (FSS). Study visits were performed at baseline, at 8, and at ±16 weeks (depending on the menstrual cycle: min–max 14.3–22.4 weeks). All study initiation and termination samplings and visits were planned 2–5 days after start of the menstrual phase or at day 2–5 of the pill-free week, since sex hormones may interact with both cortisol and vitamin D (9, 23). At these moments, saliva sampling, neurological examination, and fill-out of questionnaires were performed. Blood and urine samples were obtained at all study visits.

### Laboratory Assays

#### HPA-Axis

Activity of the HPA-axis was assessed by analyzing the CDC and the CAR. Hereto, saliva was sampled direct upon wakening followed by samples every 15 min during 1 h (CAR) and at 11 a.m., 3 p.m., 8 p.m., and 10 p.m. (CDC; nine samples in total), on two consecutive days. Saliva was collected in Salivettes^®^ Cortisol (Sarstedt, Nümbrecht, Germany) and after centrifuging (1,000 *g*, room temperature, 2 min) stored at −20°C until cortisol analyses at the end of the study. Cortisol levels were measured by liquid chromatography-tandem mass spectrometry (LC-MS/MS) as described previously (24).

#### Safety and Efficacy

At each study visit, calcium, albumin, and creatinine concentrations in blood were measured. Also, the molar calcium:creatinine urinary concentrations were determined, and considered abnormal if >1.0 (25). Analytes were measured on routine clinical chemistry analyzers (Beckman DXC880i, Beckman Coulter, Woerden, the Netherlands; Cobas 8000, Roche Diagnostics, Mannheim, Germany). Serum samples were stored at −80°C in which 25(OH)D levels were measured at the end of the study using LC-MS/MS as described previously (26). For 25(OH)D conversion between studies: 50 nmol/L = 20 ng/mL.

### Statistical Analyses

Due to non-normal distributions, continuous data are provided as medians with interquartile ranges (25th–75th percentile). Categorical data are provided as *n* (%). Comparisons of continuous variables between groups were performed using Mann–Whitney *U* tests for unpaired data, and within group comparisons were performed using Wilcoxon signed-rank tests for paired data. In addition to the non-parametric tests an ANCOVA model was used to analyze the effect of treatment on the CAR AUC, with week 16 data as dependent variable, trial medication as fixed factor, and baseline CAR, age, and 25(OH)D levels at T0 as covariates. Cortisol values were averaged for corresponding sampling times on the two consecutive days, and log transformed for assessment of circadian slopes. For each individual, AUCs for the CDC and CAR were computed using trapezoidal estimation, and the slope of the CDC was computed using linear regression, using GraphPad Prism version 7 (GraphPad Software Inc., La Jolla, CA, USA). To compare changes in HADS-D over time between groups, corrected for fatigue, ANCOVA tests were performed with week 16 data as dependent variable, trial medication as fixed factor and baseline data and FSS as covariates. A *p*-value < 0.05 was considered statistically significant. Statistical analyses were conducted with SPSS version 23.0 (IBM Corp., Armonk, NY, USA).

## Results

### Study Population

**Figure 1:**
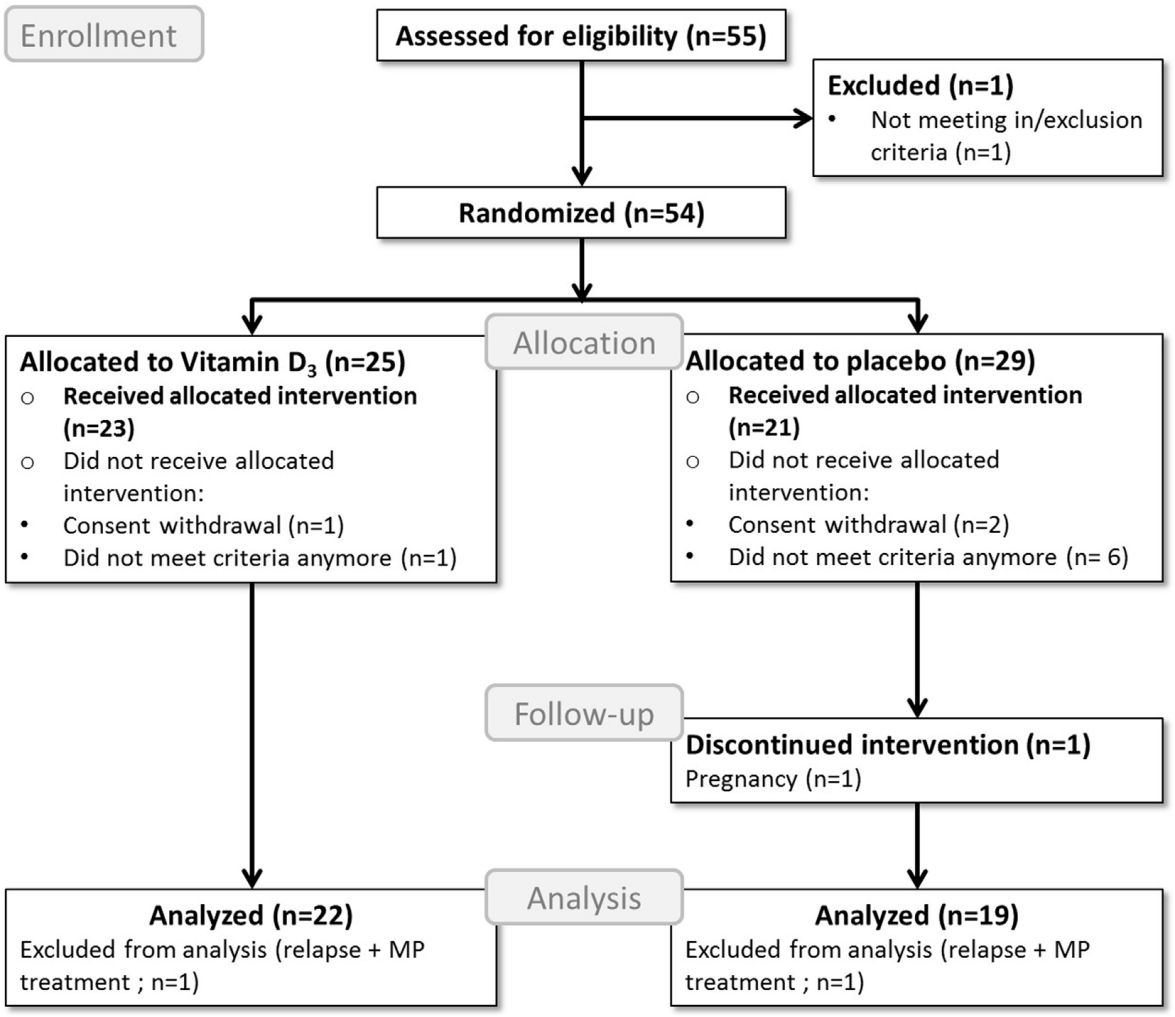
Flow diagram of the study process. MP, methylprednisolone; Analyzed = included in the primary endpoint analysis; Received allocated intervention = included in the safety analysis.

A total of 43 participants completed the study, of which 23 were allocated to the vitamin D_3_ group and 20 to the placebo group. Two participants, one participant in each group, were excluded from further analyses due to glucocorticoid treatments because of relapses, as shown in the study flow diagram (Figure 1). Baseline characteristics of the remaining 41 participants can be found in Table 1.

**Table 1:** Baseline characteristics.

	Vitamin D_3_	Placebo
Number of patients	22	19
Age, years M (Q1–Q3)	38.6 (28.0–45.0)	35.1 (33.0–45.0)
RRMS duration, years M (Q1–Q3)	3.8 (2.8–11.4)	5.4 (1.2–7.9)
Relapses in last year, *n* (%) 0 1 2	18 (82%) 3 (14%) 1 (5%)	14 (74%) 4 (21%) 1 (5%)
Duration since last relapse, years M (Q1–Q3)	2.8 (1.2–4.9)	2.6 (1.0–5.3)
EDSS score M (Q1–Q3)	2.0 (1.4–2.0)	2.0 (1.0–2.5)
HADS-D score M (Q1–Q3)	2 (1–5)	2 (1–4)
25(OH)D, nmol/L M (Q1–Q3)	85 (71–111)	78 (68–95)
Treatment, *n* (%) No DMD Interferon-beta Glatiramer acetate Dimethylfumarate Teriflunomide Fingolimod	3 (14%) 10 (45%) 0 (0%) 1 (5%) 2 (9%) 6 (27%)	1 (5%) 9 (47%) 2 (11%) 5 (26%) 0 (0%) 2 (11%)
Vitamin D supplements, *n* (%) <400 IU/day 400–800 IU/day 800–1,000 IU/day 1,000 IU/day	6 (27%) 1 (5%) 12 (55%) 3 (14%)	2 (11%) 3 (16%) 10 (52%) 4 (21%)
OC use, *n* (%) Yes No	14 (64%) 8 (36%)	7 (37%) 12 (63%)
Season of start, *n* (%) Summer (April–September) Winter (October–March)	9 (43%) 13 (57%)	11 (58%) 8 (42%)
Follow-up (weeks) M (Q1–Q3)	16.4 (16.0–17.8)	16.0 (15.4–16.9)

*M, median; Q1–Q3 = 25th–75th percentile; RRMS, relapsing-remitting multiple sclerosis; EDSS, expanded disability status scale; HADS-D, depression subscale of the hospital anxiety and depression scale; DMD, disease modifying drugs; OC, oral contraception*.

### Biological Effects and Safety of Vitamin D_3_ Supplements

**Figure 2:**
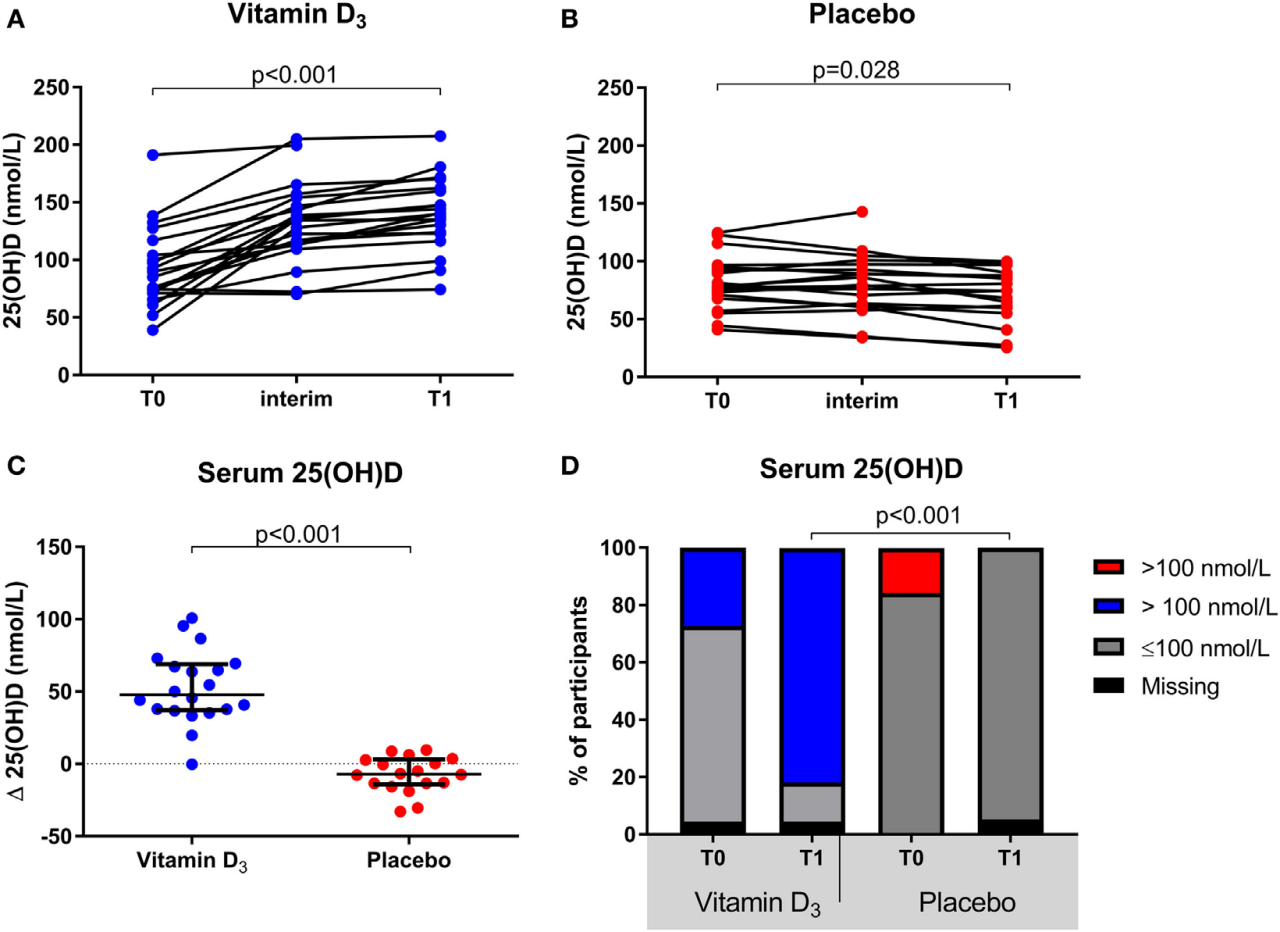
Effect of vitamin D_3_ supplements on serum 25(OH)D levels. **(A)** Within group comparisons of serum 25(OH)D levels at T0 (baseline) and T1 (week 16) in the vitamin D_3_ supplemented arm (*n* = 22), **(B)** within group comparisons of serum 25(OH)D levels at T0 and T1 in the placebo arm (*n* = 19), **(C)** between group comparisons of the serum 25(OH)D level differences between T1 and T0. **(D)** group comparisons of serum 25(OH)D levels < and >100 nmol/L at T0 and T1. *p*-Values calculated with **(A,B)** Wilcoxon signed ranks, **(C)** Mann–Whitney *U* or **(D)** χ^2^ test.

The group of participants which received vitamin D_3_ supplementation showed a significant increase in serum 25(OH)D levels at T1 [139.9 (123.5–161.2)] compared to T0 [85.0 (71.3–110.5); *p* < 0.001], whereas they slightly declined within the placebo group [77.5 (67.9–95.2) to 74.5 (58.6–88.1); *p* = 0.028; Figures 2A,B]. This resulted in a significantly higher median serum 25(OH)D level in the vitamin D_3_ group at T1 (*p* < 0.001) and the difference in change was significant between groups (*p* < 0.001; Figure 2C). Furthermore, at T1 none of the participants in the placebo group had 25(OH)D levels >100 nmol/L, in contrast to 82% of the vitamin D_3_ group (Figure 2D). Treatment with interferon-beta was not associated with a different biochemical response to vitamin D_3_ supplements (data not shown).

In each group one relapse occurred. Five participants had increased spot urine calcium:creatinine ratios at the interim visit, with three of them in the placebo group; all had normalized at the end of trial visit. No cases of hypercalcemia were observed, and the one patient with an episode of abdominal pain and hematuria suspected for a renal stone was in the placebo group. The frequency of other adverse events (summarized in Table 2), particularly infections, was similar in the two groups.

**Table 2:** Overview of adverse events in participants who received the allocated intervention.

Adverse event type	All (*n*)	Vitamin D_3_ (*n*)	Placebo (*n*)
Relapse	1	1	1
Hypercalciuria	5	2	3
Hypocalcemia	0	0	0
**Infections**			
Urinary tract infection	6	3	3
Pharyngitis	1	1	0
Ear infection	2	1	1
Herpes zoster infection	1	1	0
Influenza	3	0	3
Upper respiratory tract infection	4	1	3
**Gastrointestinal**			
Abdominal pain	1	0	1
Stomach discomfort	1	0	1
**Cardiovascular**			
Hypertension	1	0	1
Hypercholesterolemia	1	0	1
Cold extremities	1	0	1
**Musculoskeletal**			
Lumbago	1	0	1
Leg pains	1	1	0
Ruptured ankle ligaments	1	0	1
Achilles tendinosis	1	0	1
Lateral epicondylitis	1	0	1
**Other**			
Pregnancy	1	0	1
Iron deficiency	1	0	1
Headaches	2	2	0
Fatigue	2	1	1
Dizziness	2	2	0
Axillary pains	1	1	0
Globus/tingling sensations during swallowing	2	2	0
Mood swings	2	2	0
Suicidal thoughts	1	0	1

### Vitamin D_3_ Supplementation Was Not Associated With CDC, but Showed a Trend Toward a Reduction of CAR

**Figure 3:**
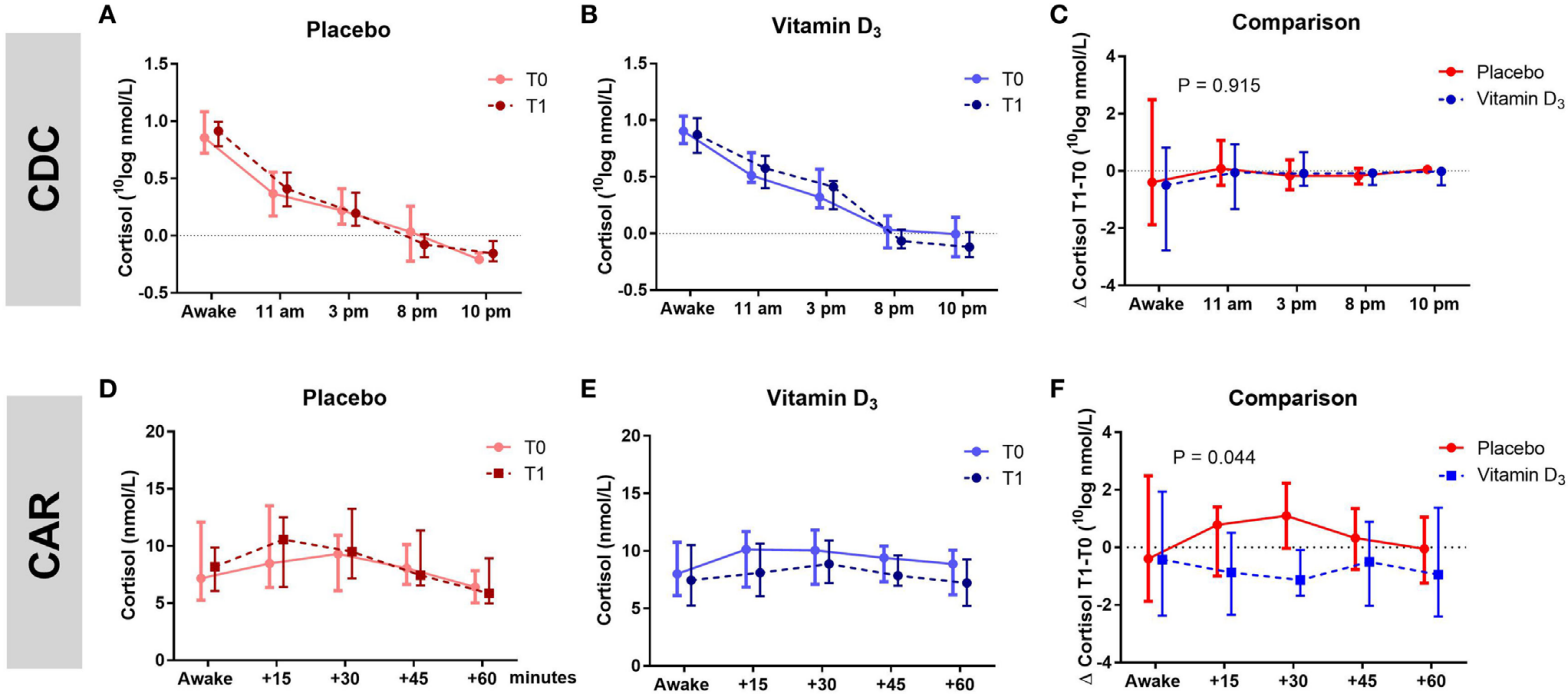
Association of vitamin D_3_ supplements with HPA-axis activity. Within group comparisons of the cortisol day curves (CDC) at T0 (baseline) and T1 (week 16) in the placebo arm [**(A)**; *n* = 19] and in the vitamin D_3_ arm [**(B)**; *n* = 21], and within group comparisons cortisol awakening responses (CAR) at T0 and T1 in the placebo arm [**(D)**; *n* = 19] and in the vitamin D_3_ arm [**(E)**; *n* = 22]. Between group comparison for the CDC [**(C)**; T1–T0] and CAR [**(F)**; T1–T0]. Data-points show median values with corresponding interquartile ranges. *p*-Value shows difference in area under the curve between vitamin D and placebo group tested with the Mann–Whitney *U* test.

The AUC of the CDC as well as the slopes of the CDC did not differ between groups (Table 3; Figures 3A–C). Within the vitamin D_3_ group, however, there was a trend toward a decrease of the AUC of the CAR [39.16 (27.41–42.07) at T0 to 33.37 (26.75–38.08) at T1; *p* = 0.095; Figure 3E] which was not seen in the placebo group Figure 3D. This resulted in a significant T1–T0 difference between the groups (*p* = 0.044; Table 3; Figure 3F). However, although not significant, numerical differences in T0 CAR AUC, 25(OH)D levels and age were observed between treatment arms. There was no significant reduction of CAR AUC at T1 when corrected for T0 CAR AUC in an ANCOVA model [*F*(1,37) = 3.601, *p* = 0.066], in which age (*p* = 0.139) and T0 25(OH)D level (*p* = 0.619) did not contribute to the model.

**Table 3:** Within and between group comparisons for circadian cortisol levels.

	Vitamin D_3_ (*n* = 22)			Placebo (*n* = 19)			
	T0 M (Q1–Q3)	T1 M (Q1–Q3)	*p*-Value*	T0 M (Q1–Q3)	T1 M (Q1–Q3)	*p*-Value*	*p*-Value**
AUC of CDC	13.11 (9.24–15.05)	11.4 (9.21–14.04)	0.357	10.14 (8.49–12.34)	9.92 (7.93–12.04)	0.344	0.915
Slope of CDC	−0.23 (−0.27 to −0.19)	−0.26 (−0.28 to −0.23)	0.159	−0.24 (−0.29 to −0.18)	−0.23 (−0.29 to −0.20)	0.778	0.347
AUC of CAR	39.16 (27.41–42.07)	33.37 (26.75–38.08)	0.095	33.90 (25.92–44.61)	35.00 (25.46–49.23)	0.260	0.044

*AUC, area under the curve; CDC, cortisol day curve; CAR, cortisol awakening response; T0 = baseline; T1 = week 16; M = median; Q1–Q3 = 25th–75th percentile*.

*p-Value T0 vs. T1 within each group was calculated with the Wilcoxon Signed ranks test.

**Between group comparisons of T1–T0 differences were tested with the Mann–Whitney U test.

No difference between participants treated with interferon-beta and other participants was observed (data not shown). HADS-D scores for depressive symptoms, which were low in both groups, and FSS fatigue scores, showed no significant differences between groups at both T0 and T1, and did not significantly change over time (Table 4).

**Table 4:** Within and between group comparisons for depression and fatigue scores.

	Vitamin D_3_ (*n* = 22)			Placebo (*n* = 19)			
	T0 M (Q1–Q3)	T1 M (Q1–Q3)	*p*-Value*	T0 M (Q1–Q3)	T1 M (Q1–Q3)	*p*-Value*	*p*-Value**
HADS-D Score	2.0 (1.0–5.0)	2.0 (1.0–5.0)	0.662	2.0 (1.0–4.0)	3.0 (1.0–4.0)	0.904	0.714
FSS Score	5.0 (3.7–5.8)	4.6 (3.9–6.1)	0.578	4.3 (3.8–5.8)	4.3 (3.4–5.2)	0.255	0.946

*HADS-D, depression subscale of the hospital anxiety and depression scale; FSS, Fatigue Severity Scale*.

*p-Value T0 vs. T1 within each group was calculated with the Wilcoxon Signed ranks test.

**Between group comparisons of T1–T0 differences were tested with the Mann–Whitney U test.

## Discussion

In this prospective controlled study among female patients with RRMS, we assessed the association of vitamin D_3_ supplements with the activity of the HPA-axis. In both groups, there were no significant changes in salivary cortisol levels across the day, but a trend toward a reduction in CAR was observed in the vitamin D_3_ supplemented compared to the placebo group. These findings suggest CAR as the most sensitive marker to investigate a suppression of HPA-axis activity in non-depressed MS patients by vitamin D_3_ supplements. Further studies should consolidate this finding and assess whether it has relevant implications for the disease course of MS.

Hyperactivation of the HPA-axis has been observed in MS (16, 27). This has been assessed mostly by the dexamethasone suppression test often followed by CRH-stimulation (Combined Dex/CRH-test) (13, 15), or post-mortem assessments of hypothalamic CRH-neurons (28, 29). These methods have shown increased HPA-activity in progressive MS, but not or less pronounced in RRMS (13, 15). However, increased CAR has been observed in RRMS, but not or less pronounced in progressive MS (12, 16). Intriguingly, increased CAR in RRMS has been associated with increased disability progression (16), which makes this a relevant outcome to be suppressed by vitamin D_3_ supplements. Kern et al. argue that different aspects of HPA-activity are investigated by the Dex/CRH-test and circadian cortisol levels (16). The Dex/CRH-test may reflect dysregulated feedback, possibly related to lesions or neurodegeneration, whereas increased CAR may indicate compensation for immune activation. In this light, our study addresses a relevant model of cortisol and vitamin D as two anti-inflammatory nuclear receptor ligands within a network (9), which may be exploited as a whole to provide benefit for patients suffering from MS. Likewise, Mirhosseini et al. recently showed in healthy controls an interaction between serum 25(OH)D levels and another nuclear receptor ligand, thyroid hormone, in which 25(OH)D levels exceeding 125 nmol/L were associated with optimal thyroid function (30).

Another relevant cortisol-related outcome in MS patients could be the risk of depressive symptoms. In MS, we observed associations between depressive symptoms and low 25(OH)D levels (7, 31), Despite well-known immune regulatory properties of vitamin D *in vitro* and in animal models, we previously did not observe signals suggestive of an effect of vitamin D supplements on depression related immunological outcomes in an underpowered study among non-depressed RRMS patients (32). Our similar current cohort does not allow us to make statements on depressive symptoms either. However, a suppressed CAR could associate with a lower risk of developing depressive symptoms. A high CAR is an important predictor of depressive episodes (33, 34), and also has been associated with depression in MS (35).

This study has several limitations. First, due to difficulties with inclusion, we did not reach the originally projected sample size and can only show associations in a small dataset. Although no clear significant effects were present for AUC of CAR and CDC and slope of the CDC comparing the groups, the final sample size may have been too small to detect small but significant effects of vitamin D_3_ supplementation. Second, the participants in this study had low depression scores. Whether similar results can be obtained in depressed participants with high depression scores, remain to be seen. Strengths of the study are the placebo-controlled design and the standardized measurements. We only included female pre-menopausal patients, and all measurements were performed in a standardized phase of the menstrual cycle, minimizing influence of hormonal status. Furthermore, the vitamin D_3_ dose supplemented induced 25(OH)D levels exceeding 100 nmol/L in most patients, which has been advocated by a recent ECTRIMS workgroup report to be beneficial for MS related outcomes (36).

Altogether, our study identified a trend toward reduction of CAR as an appropriate outcome to investigate suppression of the HPA-axis by vitamin D_3_ supplements in non-depressed MS. This interaction could be relevant for the evolution of MS related outcomes, such as (early) progression of disability or development of depressive symptoms. Further studies should consolidate this finding and assess its relevance for patients with MS.

## Ethics Statement

The study, with the amendments, was approved by the local ethical research committee “METC-Z” (NL45995.096.14/14-T-75), and written informed consent was obtained from all study participants. The trial was registered on ClinicalTrials.gov (NCT02096133) and EudraCT (2014-000728-97). The study was performed in accordance with the Declaration of Helsinki and guidelines for Good Clinical Practice.

## Author Contributions

IH, JD, JS, and RH contributed conception and design of the study; DK, JO, and LR contributed to data collection and validation; LR performed the statistical analysis and wrote the first draft of the manuscript. All authors contributed to manuscript revision, read, and approved the submitted version.

## Conflict of Interest Statement

LR, JD, IH, DK, and JO report no disclosures. JS received lecture and/or consultancy fees from Biogen, Merck, Sanofi-Genzyme, and Novartis. RH received honoraria for lectures and advisory boards, and/or Institutional and Research Grants from Merck, Biogen, Sanofi-Genzyme, Novartis, and TEVA.
